# Activities of starch synthetic enzymes and contents of endogenous hormones in waxy maize grains subjected to post-silking water deficit

**DOI:** 10.1038/s41598-019-43484-0

**Published:** 2019-05-07

**Authors:** Huan Yang, Xiaotian Gu, Mengqiu Ding, Weiping Lu, Dalei Lu

**Affiliations:** Jiangsu Key Laboratory of Crop Genetics and Physiology/Jiangsu Key Laboratory of Crop Cultivation and Physiology, Agricultural College of Yangzhou University/Jiangsu Co-Innovation Center for Modern Production Technology of Grain Crops, Yangzhou, 225009 China

**Keywords:** Seed development, Drought

## Abstract

Rainfed maize in Southern China and frequently suffer water deficit at later plant growth periods. A pot trial in 2014–2015 was conducted to study the effects of drought stress (the relative soil moisture contents are 70–80% and 50–60% under control and water deficit conditions, respectively) after pollination on grain filling and starch accumulation, activities of starch synthetic enzymes, and contents of indole-3-acetic acid (IAA) and abscisic acid (ABA), with Suyunuo5 as test material. The grain fresh weight, volume, and dry weight were not affected by drought before 10 days after pollination but were restricted thereafter. The reduction at maturity was reduced by 33.3%, 40.0%, and 32.3% in 2014 and by 21.7%, 24.3%, and 18.3% in 2015. The grain filling rate was suppressed by water deficit, whereas grain moisture and starch content were slightly affected. The starch accumulation was decreased by 33.5% and 20.0% at maturity in 2014 and 2015, respectively. The activities of starch synthetic enzymes (sucrose phosphate synthase, sucrose synthase, ADP-glucose pyrophosphorylase, soluble starch synthase, and starch branching enzyme) were downregulated by post-silking drought. The ABA content was increased, whereas IAA content was decreased when plants suffered water deficit during grain filling. In conclusion, post-silking water deficit increased ABA content, decreased IAA content, and weakened the activities of starch synthetic enzymes, which suppressed grain development and ultimately reduced grain weight.

## Introduction

Drought stress is a main environmental constraint that restrict crop productivity worldwide. Maize are prone to drought as they are mostly grown under rainfed conditions. The annual loss of maize yield caused by water deficit dominates all environmental stresses, and the incidence is acute with increasing intensity and frequency^1^.

Grain filling duration and rate are crucial to final grain weight^2^. Studies have demonstrated that drought stress at early grain development disturb the cell division and differentiation processes required for organ establishment and starch biosynthesis^3,4^. Starch is the foremost grain component that determines grain weight, and subject to water deficit after flowering stage is, in most cases, associated with the reduced starch accumulation^5^. Water deficit occurring at initially grain development stage curtails the grain sink potential by reducing the number of endosperm cells and amyloplasts formed, thus reducing grain weight by decrease the endosperm cells to deposite starch, in terms of both duration and rate^6,7^. Studies reported that postanthesis mild drought stress shortened the grain filling period and increased grain filling rate and starch accumulation rate by enhancing the activities of starch synthetic enzymes (sucrose synthase (SuSy), ADP Glc pyrophosphorylase (AGPase), soluble starch synthase (SSS), and starch branching enzyme (SBE)), whereas the activities of those starch synthetic enzymes were weakened under severe drought stress^8–10^. The downregulation of AGPase activity at both the protein and transcript levels under water deficit conditions during grain filling is responsible for the significant decrease in starch deposition^11,12^. The sorghum grain filling rate was reduced by water shortage at the flowering stage due to the reduced activities of SSS, SBE, and granule-bound starch synthase^13^.

The grain endogenous hormones such as indole-3-acetic acid (IAA) and abscisic acid (ABA) significantly affect the rate and duration of grain filling^14^. Drought stress during grain filling increases the ABA content, which induces the increase of the rate and early suspension of duration^15^. Yang and Zhang^2^ found that high grain ABA content under moderately soil-dried condition increased the rate, whereas too high ABA content under severely soil-dried condition reduced the rate of grain filling. Application of exogenous ABA at middle grain filling stages could regulate sink activity, grain weight was noticeably reduced with high ABA spraying concentration and mildly increased with low concentration^16^. Guoth *et al*.^17^ found that drought-tolerant wheat genotypes only exhibited higher ABA content at early grain filling, and the sensitive cultivars maintained high ABA levels in the later grain filling stages, which led to a decreased grain yield. Lv *et al*.^18^ and Liu *et al*.^19^ observed that post-anthesis water stress decreased the wheat grain weight and grain filling rates due to the decrease of grain IAA and ABA contents at the early and middle stages of grain filling. Our early studies reported that waxy maize flour and starch physiochemical properties were changed by drought after pollination^20,21^. Starch formation and accumulation were co-adjusted by a series of starch synthetic enzymes and endogenous hormones, and clarfying the effects of post-silking water deficit on those enzymes and hormones could improve our understanding on starch development under drought conditions. In the present research, the grain filling and starch accumulation were clarified, the activities of starch synthetic enzymes and contents of endogenous hormones (ABA and IAA) in response to post-silking water deficit were studied. The results may offer a reference for drought-stressed starch formation of rainfed waxy maize that undergo water deficit after silking.

## Results

### Grain filling

**Figure 1:**
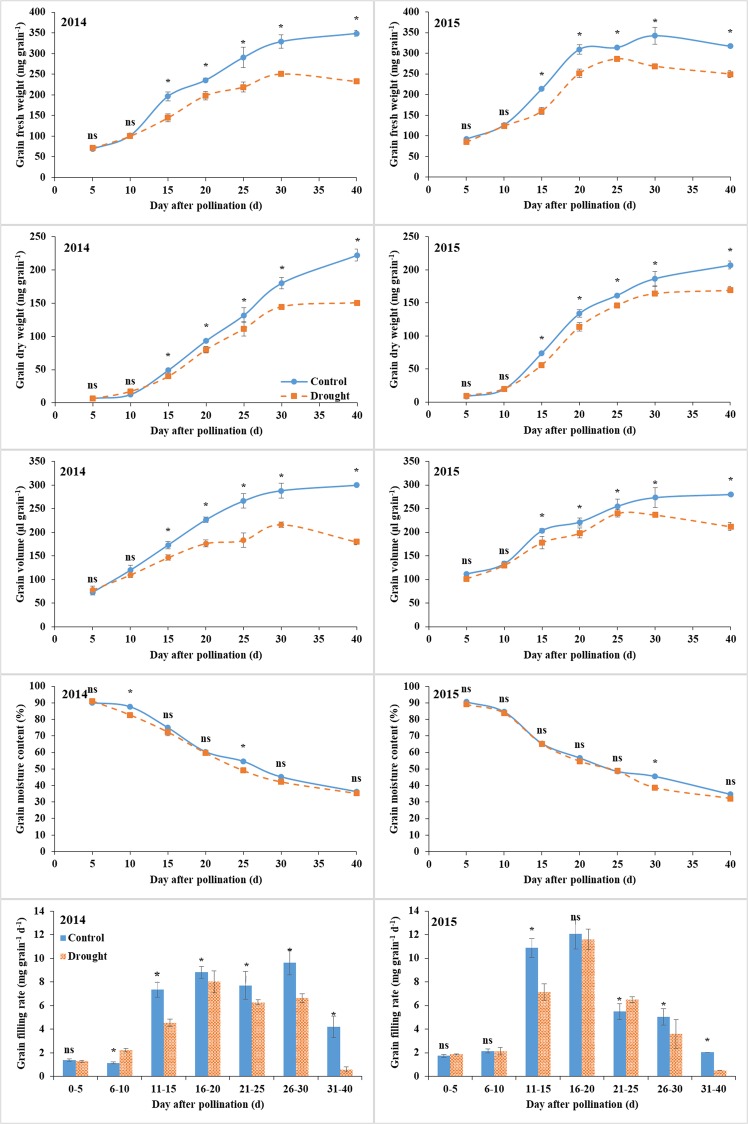
Grain filling properties (fresh weight, fresh volume, dry weight, water content, and filling rate) under control and water deficit conditions. Bars are standard errors of three replicates. *Significant at *p* < 0.05 level; ns, not significant.

Water deficit did not affect the grain fresh weight, volume, and dry weight before 10 days after pollination (DAP), but they were restricted thereafter. The reduction gradually increased with grain development. At maturity (40 DAP), the grain fresh weight, volume, and dry weight were reduced by 33.3%, 40.0%, and 32.3% in 2014 and by 21.7%, 24.3%, and 18.3% in 2015, respectively (Fig. 1). The grain moisture content was slightly affected by drought, whereas the reduction was significantly affected at 10 and 25 DAP in 2014 and 30 DAP in 2015, and not affected at the other stages. The grain filling rate in 2014 was similar to control before 5 DAP, increased by drought at 6–10 DAP, and decreased thereafter. The value in 2015 was not affected by drought before 10 DAP, decreased at 11–15 DAP, not affected at 16–20 DAP, increased at 21–25 DAP, and decreased thereafter.

### Starch accumulation

**Figure 2:**
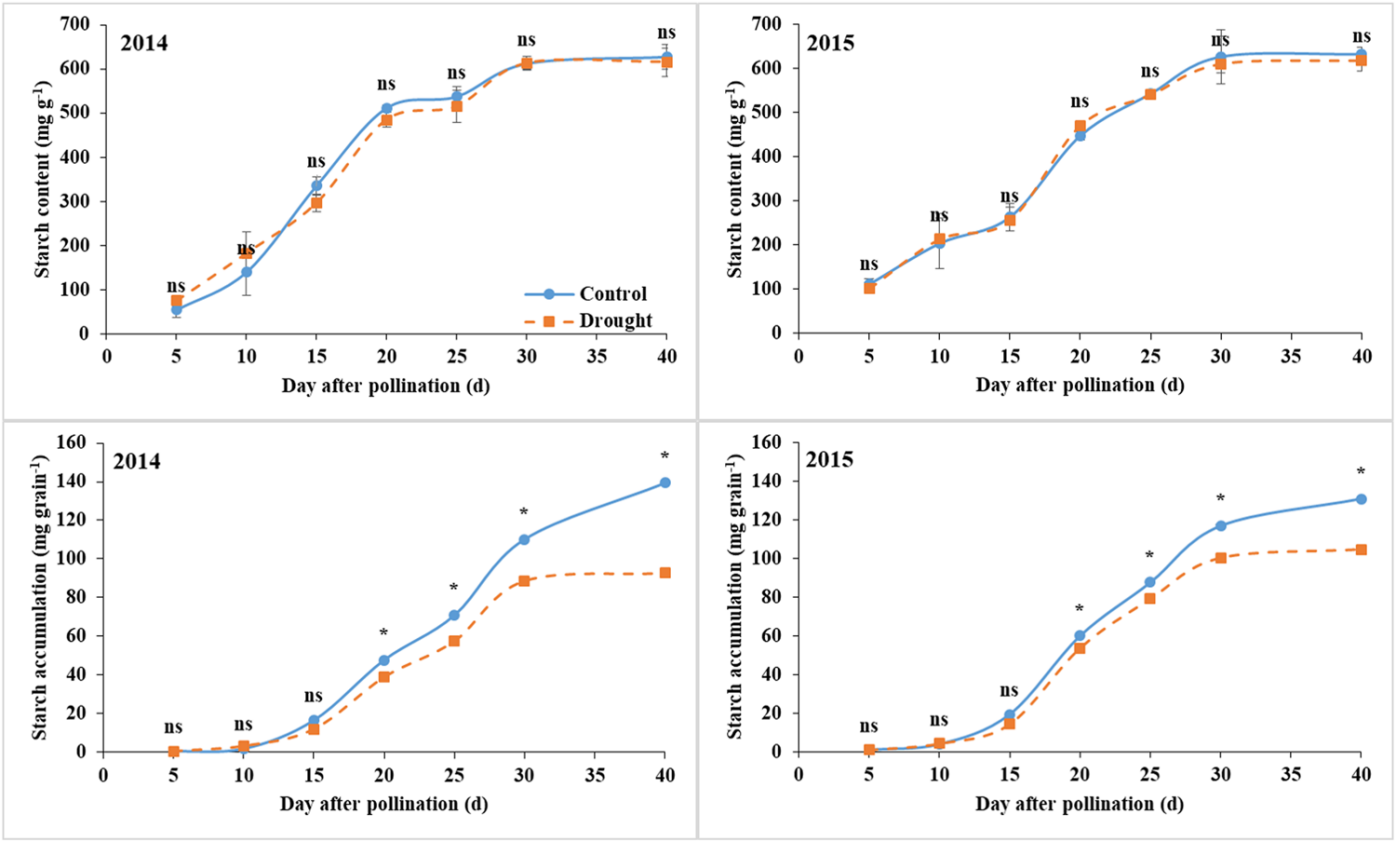
Grain starch concentration and accumulation under control and water deficit conditions. Bars are standard errors of three replicates. *Significant at *p* < 0.05 level; ns, not significant.

The grain starch concentration (mg g^−1^) was slightly affected by water deficit throughout the grain filling in both years (Fig. 2). The starch accumulation (starch content × grain dry weight, mg grain^−1^) was not affected by drought before 15 DAP, and the value was reduced by water deficit thereafter, similar to the reduction of grain dry weight. At maturity, the reduction of starch accumulation under drought was 33.5% and 20.0% in 2014 and 2015, respectively.

### Activities of SPS and SuSy

**Figure 3:**
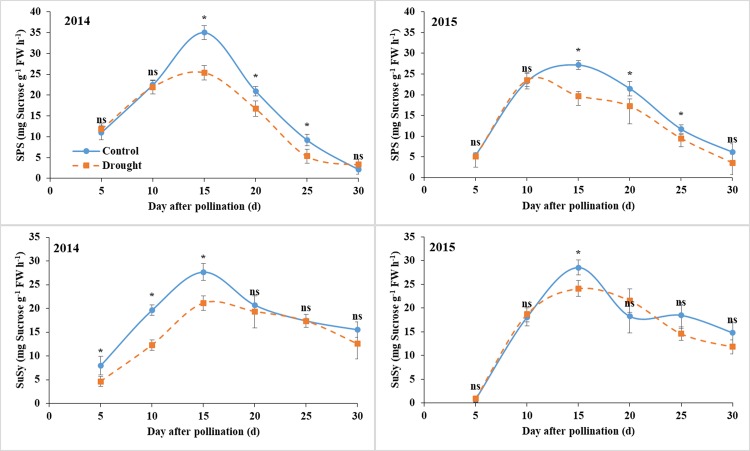
Grain SPS and SuSy activities under control and water deficit conditions. Bars are standard errors of three replicates. *Significant at *p* < 0.05 level; ns, not significant.

The grain SPS activity was not affected by drought at 5–10 DAP and decreased at 15–25 DAP, and the difference disappeared at 30 DAP in both years (Fig. 3). The SuSy activity in 2014 was decreased by drought at 5–15 DAP and not affected thereafter, the value in 2015 was only reduced by drought at 15 DAP and was similar to drought at the other stages. In general, the average reductions of SPS and SuSy activities throughout the grain filling were 16.1% and 19.8% in 2014 and 17.3% and 7.2% in 2015.

### Activities of AGPase, SSS, and SBE

**Figure 4:**
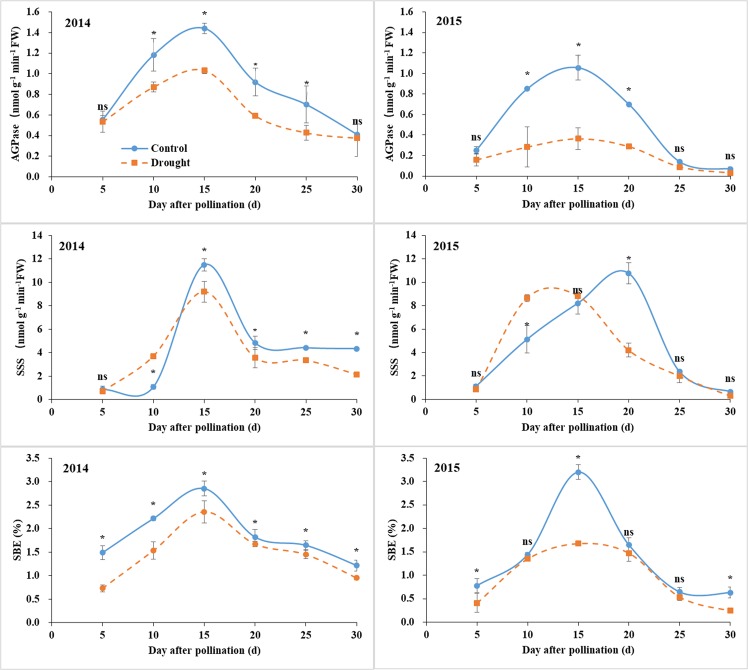
Grain AGPase, SSS, and SBE activities under control and water deficit conditions. Bars are standard errors of three replicates. *Significant at *p* < 0.05 level; ns, not significant.

The activities of starch synthetic enzymes (AGPase, SSS, and SBE) were significantly influenced by post-silking drought (Fig. 4). The AGPase activity in both years was decreased by drought, and the decrease was pronounced at 10–25 DAP in 2014 and 10–20 DAP in 2015. The SSS activity in 2014 was similar to control at 5 DAP, increased at 10 DAP, and decreased thereafter. The value in 2015 was not affected by drought at 5, 15, and 25–30 DAP; increased at 10 DAP; and reduced at 20 DAP. The SBE activity in 2014 was reduced by drought throughout the grain filling, and the value in 2015 was also reduced by drought at 5, 15, and 30 DAP and was not affected at 10 and 20–25 DAP.

### ABA and IAA content

**Figure 5:**
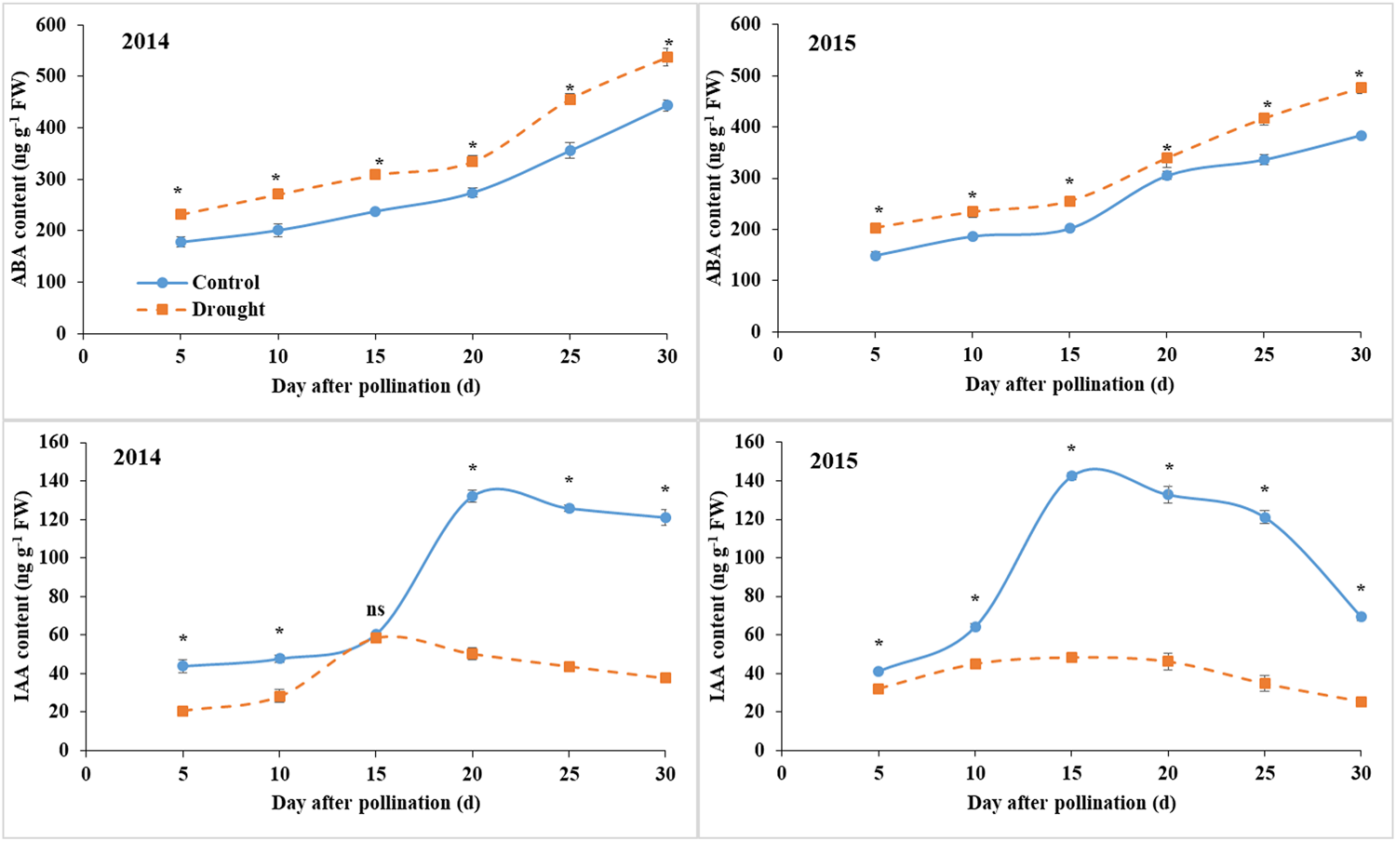
Grain ABA and IAA contents under control and water deficit conditions. Bars are standard errors of three replicates. *Significant at *p* < 0.05 level; ns, not significant.

The grain ABA content gradually increased with grain development, and it was increased by drought throughout the grain filling (Fig. 5). The IAA content in 2015 was decreased by drought, the value in 2014 was not affected at 15 DAP and reduced at the other stages, and the decrease was severe after 15 DAP in both years.

## Discussion

In the present study, the grain weight (fresh or dry), volume, and average grain filling rate were decreased by post-silking drought stress. The severe reduction of in 2014 may be due to the high temperature and low sunlight duration (Post-silking day/night temperature and sunlight were 29.0/22.0 °C and 126 h in 2014 and were 28.2/21.5 °C and 145 h in 2015, respectively. Data available on http://www.tianqihoubao.com/lishi/yangzhou.html), as those heat and sunlight stresses also reduce grain weight^1^. The small volume and low weight of grains under water stress may be caused by the reduced endosperm cell numbers and few formed amyloplasts, which induced the decrease of grain sink potential^6^. The reduced rate of grain filling under drought condition induced the low grain weight, as it could serve as an indirect selection criterion for grain filling rate^22^. Grain moisture content declines during the entire grain filling, and final grain weight is achieved at values closed to 35% and moderate stress conditions affecting plant development seem to have little impact over this value^23,24^. The similar grain moisture content under control (36.3% and 34.7% in 2014 and 2015) and drought (35.3% and 32.2% in 2014 and 2015) conditions indicated semblable grain filling progress, similar observations were also reported on fresh waxy maize^21^ and normal maize^25^, while extreme drought stress shortened this duration^25^. Studies on wheat and rice revealed that mild drought after anthesis shortened the duration but increased the rate of grain filling, whereas severe water shortage reduced both the rate and duration^8–10^. The possible explanation for this phenomenon may be due to the similar reduction of both source and sink potential in response to post-silking water deficit^21^, and the value mainly different among genotypes and little affected by moderate stresses^23,24^. Therefore, further study different water stress levels on maize grain filling may help clarify this different response.

Grain development is dependent on many factors, including sucrose availability and the activities of enzymes involved in grain starch and sugar metabolism^26^. Drought during grain filling restricted the starch deposition were reported in many crops^5^. We observed that the grain starch concentration was not affected (only reduced by 1.8% and 2.2% at maturity in 2014 and 2015, respectively), but the starch accumulation was severely depressed (reduction was 33.5% and 20.0% at maturity in 2014 and 2015, respectively) by post-silking water deficit, and the severe reduction of starch accumulation in 2014 may be due to the severe reduction of grain dry weight (32.3% and 18.3% in 2014 and 2015, respectively). Similar starch concentration between control and drought stress was observed on fresh waxy maize^21^ and normal maize^27^. A field study on normal maize^28^ and sorghum^29^ also observed that starch content was slightly affected by restrict irrigation. The starch accumulation was remarkably reduced by drought, which may caused by the weakened activities of starch synthetic enzymes (SPS, SuSy, AGPase, SSS, and SBE), this opinion was also demonstrated at protein, transcript, and enzymology levels when plants suffered drought at late growth stages in wheat^10–12^ and sorghum^13^.

ABA and IAA are two important endogenous hormones that significantly affect the grain endosperm cell division and expansion, grain size initiation, and grain filling rate and duration^30^. High IAA content can promote endosperm cell division and increase sink potential, which accelerate endosperm cell propagation and grain filling^31,32^. ABA is involved in the metabolic activity of key enzymes in sucrose decomposition and starch biosynthesis and regulates the process of grain weight formation^16^. Drought stress increases the amounts of ABA and affects the embryo mutation, grain development, and grain components^33–35^. The reduced IAA content under drought was also observed in rice and caused spikelet sterility^36^. In the present study, the increased ABA content and decreased IAA content under post-silking drought stress restricted grain filling, resulted the low grain weight, which may be due to drought restricted the endosperm cell division, grain sink size initiation, filling capacity, and grain weight^16,30,35,37^.

## Conclusion

Post-silking water deficit decreased the grain weight (dry or wet), volume, and filling rate, and the severe reduction of those parameters in 2014 may be due to the high temperature and low sunlight duration at grain filling stage. The grain moisture content and starch concentration were slightly affected by drought stress, whereas the starch deposition was severe restricted due to the severe decrease of grain weight. The reduction of starch deposition in grains was caused by the weakened activities of starch synthetic enzymes (SPS, SuSy, AGPase, SSS, and SBE), reduced IAA content and increased ABA content.

## Materials and Methods

### Plant materials and growth conditions

Suyunuo5, a well-known waxy maize covering large plantation areas in Southern China, was analysed in farm at Yangzhou University (Yangzhou, China) in 2014 and 2015. Seeds were sown in March 15 and transplanted to plastic pots (two seedlings per pot, with one plant retained at the jointing stage) in March 28. Each plastic pot was 38 cm in height and 43 cm in diameter, and loaded with 30 kg of sieved sandy loam soil. The plants per pot were provided 10 g compound fertilizer (N/P_2_O_5_/K_2_O = 15%/15%/15%) at transplantation and 6.6 g urea (N = 46%) at the jointing stage.

Plants were maintained up to silking stage under relative soil moisture content of approximately 70–80%. After manual pollination at same day, plants were imposed to water deficit treatment till maturity. The relative moisture contents of the soil subjected to the control and drought treatments were 70–80% and 50–60% by the weighing method. The evaporation of the plants was supplied at each morning by measuring the weight loss. The rainfall was excluded by a mobilizable transparent waterproof canopy. Each treatment has fifty pots.

### Samples preparation

Three ears were harvested at 5, 10, 15, 20, 25, 30 DAP and maturity (40 DAP). Approximately 50–60 grains at middle position were stripped from the ears and immediately frozen in liquid N_2_, and then stored at −75 °C until analysis. 100-grains randomly selected from the left grains on independent ear were weighed (fresh weight, mg grain^−1^) and the volume were measured by drainage (fresh grain volume, μl grain^−1^). After that, the grains were deactivated at 105 °C for 30 min and dried at 60 °C to consistent weight. Then, the grain dry weight (mg grain^−1^) and grain moisture content (%) was determined based on three independent ears as triplicates. The grain filling rate (mg grain^−1^ d^−1^) was calculated based on the dry weight difference between two contiguous sampling periods. After drying and weighing, the grains were pulverized and passed through a filter screen (100-mesh, d = 0.149 mm) for starch content determination.

### Starch analysis

The grain starch content (mg g^−1^) was determined using the anthrone-sulfuric acid method^38^. Starch accumulation (mg grain^−1^) was determined using the following formula: grain dry weight × starch content.

### Assays of SPS and SuSy activities

All procedures for enzyme extraction were carried out in ice (0 °C–2 °C). The grain embryo and pericarp were discarded, and approximately 1 g fresh weight of the lyophilized samples were homogenized in the extraction buffer containing 50 mM HEPES-NaOH (pH 7.5) followed by centrifugation at 10,000 × *g* for 10 min^39^. The supernatant was used for enzyme assays. SuSy (EC 2.4.1.13) and SPS (EC 2.4.1.14) assay followed the method proposed earlier^39^ with modifications by Yang *et al*.^40^.

### Assay of starch synthetic enzymes

The enzyme extracting solution was prepared following a procedure that detailed by Yang *et al*.^40^. The assays of the AGPase (EC 2.7.7.27), SSS (EC 2.4.1.21) and SBE (EC 2.4.1.18) followed the method proposed by Nakamura *et al*.^41^ with slight modifications^40^.

### Endogenous hormone

The contents of endogenous hormones (IAA and ABA) in grains at different stages (5, 10, 15, 20, 25, and 30 DAP) were examined by enzyme-linked immunosorbent assay (ELISA). The IAA and ABA were extracted according to the method proposed by Lv *et al*.^18^. A plant hormone ELISA kit was purchased from Shanghai Jining Shiye Co., Ltd. (Shanghai, China), and IAA and ABA were measured according to the kit’s protocols. The recovery rates for IAA and ABA were 84.7% ± 3.2% and 91.2% ± 2.6%, respectively.

### Statistical analysis

The data are expressed as averages of triplicates. Data were subjected to ANOVA with the *LSD* test at *p* < 0.05 level using the Data Processing System (version 7.05).
